# An interpretability heart disease prediction model based on stacking ensemble with SHAP

**DOI:** 10.3389/fmolb.2025.1763157

**Published:** 2026-02-20

**Authors:** Yanjie Chen, Liqiang Chong, Zhenghao Bao, Shaoqiang Wang, Yuchen Wang, Yanan Feng

**Affiliations:** 1 Department of Abdominal Ultrasound, The Affiliated Hospital of Qingdao University, Qingdao, China; 2 Department of Urology, The Affiliated Hospital of Qingdao University, Qingdao, China; 3 School of Information and Control Engineering, Qingdao University of Technology, Qingdao, China; 4 School of Science, Qingdao University of Technology, Qingdao, China

**Keywords:** classifier, heart disease, interpretability, SHAP, stacking ensemble

## Abstract

**Introduction:**

In the big data era, healthcare data has grown exponentially, presenting opportunities to explore the pathogenesis of heart disease. Clarifying the correlations between health indicators and heart disease is crucial for early prevention. This study employs ensemble learning to identify the key influencing factors, assisting clinicians in understanding the pathogenesis and enhancing prediction strategies.

**Methods:**

A two-layer stacking ensemble model is proposed, integrating Naive Bayes, Decision Trees, CatBoost and Gradient Boosting Trees to enhance prediction accuracy. To address ensemble models’ complexity and poor interpretability, the SHAP technique is introduced to visualize the decision-making logic of the ensemble model.

**Results:**

Experimental results show that the stacking model achieved 86.69% accuracy, 87.14% weighted precision, 86.69% weighted recall, and 86.91% weighted F1-score. It balances precision and recall, unlike single learners that prioritize one over the other. Global interpretive analysis demonstrates that age, sleep duration, self-rated health status and BMI are critical factors in assessing cardiovascular risk. Local interpretive analysis is conducted to evaluate the contribution of each feature to the prediction results of individual samples.

**Discussion:**

The stacking model’s superior performance demonstrates that ensemble learning can overcome the limitations of single learners. Additionally, key predictive factors are identified: maintaining an average sleep duration of 7-8 hours significantly reduces heart disease risk, while advanced age and poor health status increase susceptibility. This study provides a reliable predictive tool for personalized heart disease prevention and treatment.

## 1 Introduction

Nowadays, heart-related diseases are the leading cause of death worldwide. A World Health Organization report estimates that cardiovascular diseases account for approximately 17.9 million deaths globally each year (Martin et al., 2024). Accurate and reliable detection of heart disease is crucial for preventing complications and enhancing cardiovascular safety. Early prediction of heart diseases can help avert or mitigate their impacts, thereby reducing mortality rates. Conventional heart disease diagnostic techniques such as reviewing patients’ medical histories, analyzing physical examination results, and evaluating relevant physiological systems—are often costly and computationally intensive (Khan et al., 2024), particularly in regions lacking state-of-the-art technologies and qualified medical professionals (Yash et al., 2024).

Medical organizations collect data on various health-related issues, which was then utilized to gain valuable insights through diverse machine learning techniques. Some researches (Pahwa and Kumar, 2017; Chandrasekhar and Peddakrishna, 2023; Ba et al., 2023; Mahesh et al., 2022; Srinivasu et al., 2024; Rimal and Sharma, 2023; Albert et al., 2023; Dalal et al., 2023; Khan et al., 2023; Yongcharoenchaiyasit et al., 2023) were carried out to make predictions more accurate, aiming to save the lives of many precious individuals. For example, to improve the prediction accuracy of models, Pahwa and Kumar (2017) used random forest and Naive Bayes model to predict heart disease and proposed a new feature selection method that combines the gain ratio and the recursive feature elimination SVM algorithm. This method assigns weights to each feature, removes features with low value, and allows high-value features to have a greater influence on disease prediction. Chandrasekhar and Peddakrishna (2023) employed six machine learning algorithms—random forest, KNN, logistic regression, Naive Bayes, Gradient Boosting Decision Tree(GBDT), and AdaBoost—to perform predictions on two public datasets: the Cleveland dataset and the IEEE Dataport dataset. Results showed that logistic regression achieved 90.16% accuracy on the Cleveland dataset, while AdaBoost attained 90% accuracy on the IEEE Dataport dataset. Ba et al. (2023) developed an early detection model for heart disease. During preprocessing, missing values were filled using mean interpolation. GBDT model incorporating all features was trained, followed by SHapley Additive exPlanations (SHAP) analysis to identify the most significant contributors to the outcomes. The final features were selected based on SHAP values exceeding 0.1. Mahesh et al. (2022) employed the AdaBoost ensemble algorithm and K-fold cross-validation, supplemented by SMOTE to address data imbalance and noise. The results demonstrated that the model achieved the highest accuracy rate of 95.47%. Srinivasu et al. (2024) constructed five models including random forest, XGBoost, logistic regression, SVM and artificial neural network to predict heart disease and verified the harm of data leakage caused by sampling on the complete dataset. Rimal and Sharma (2023) employed different optimization techniques, such as Bayesian optimization, optuna optimization, and GA search, utilizing 5 and 10 generations with Random Forest and SVM. They achieved the highest accuracies of 86.6%, 89%, and 90% respectively.

Machine learning models are prone to overfitting. In contrast, deep learning has been extensively explored and validated for its effectiveness in heart disease diagnosis (Ahmad et al., 2023; Liu et al., 2018; Shaheen et al., 2024; Ali et al., 2020; Alqahtani et al., 2022; Medina-Inojosa et al., 2024). Ahmad et al. (Ahmad et al., 2023) proposed a hybrid deep learning model based on Bidirectional Long Short-Term Memory (BLSTM) for cardiovascular disease prediction. Recursive Feature Elimination (RFE) was employed to select optimal features. The proposed hybrid model achieved an accuracy of 94.507% and an F1-score of 94%. Liu et al. (2018) developed a CNN-RNN hybrid model, which achieved a promising F1-score of 93.6% in the early prediction of acute myocardial infarction by fusing ECG signal sequences and clinical imaging features. While the hybrid architecture validates the effectiveness of multimodal feature fusion for cardiovascular disease diagnosis, a more robust and clinically interpretable solution can be achieved by leveraging ensemble strategies combined with artificial intelligence. Ensemble models integrated with explainable technologies enable healthcare professionals to rapidly identify heart disease patients, facilitating early diagnosis and intervention to reduce cardiovascular risks.

In this manuscript, we investigate the effectiveness of various machine learning algorithms and implement different ensemble techniques to improve the accuracy of heart disease prediction. Specifically, we utilize four machine learning methods—Naive Bayes (NB) (Liu and Zhang, 2020), Categorical Boosting (CatBoost) (Zhang et al., 2020), Decision Tree (DT) (Jabeur et al., 2021), and Gradient Boosting Tree (GB) (Friedman, 2001) to train and evaluate models, derive actionable insights, visualize results, interpret outcomes using SHAP (Lundberg and Lee, 2017; Lundberg et al., 2020; Ponce-Bobadilla et al., 2024), and achieve optimal performance in heart disease prediction. Experimental results demonstrate that our proposed model achieves higher accuracy than all baseline classifiers. The following are the findings of this research:

1. We propose a stacking ensemble model using NB, CatBoost, DT, and GB for the prediction of heart disease.
2. We perform soft voting and compare the results with our proposed stacking model.
3. An explainable technique, SHAP is used to understand the contribution of features in the heart disease predictions of our proposed model.

## 2 Methods

### 2.1 Base classifier

Naive Bayes (NB) Classifier operates based on Bayes’ theorem, which quantifies the probability of a class label given a set of input features. The “naive” characteristic of the algorithm stems from its core assumption: all features are conditionally independent of one another when the class label is known. The classifier first learns from labeled training data to compute prior probabilities and class-conditional probabilities. When predicting the class of a new sample, the NB classifier calculates the posterior probability of the sample belonging to each possible class using the learned probabilities, and then assigns the sample to the class with the highest posterior probability.

Decision Tree (DT) is a tree-like structure that embodies a sequential decision-making process. It starts with a root node, and recursive partitioning proceeds until all training samples within a node belong to the same class. There are various types of DTs, including ID3, C4.5, ID5.0, and CART, each based on different attribute selection criteria. A key challenge in DT construction is determining the optimal attribute for both the root node and its sub-nodes. ID3 is one of the most widely used DT variants, which employs Information Gain (IG)—a metric derived from the concept of entropy—as its attribute selection measure.

Gradient Boosting (GB) consists of three core components: an additive model, a loss function, and weak learners. The fundamental idea of GB is to combine multiple weak learners to form a strong predictive model. In each iteration, each subsequent weak learner learns from the errors of the previous one, thereby reducing the overall prediction error. This error correction is achieved by updating weights at each step, and the iterative process continues until the loss function is minimized.

CatBoost is a gradient boosting algorithm specifically designed to handle categorical data efficiently. It adopts ordered boosting to process categorical features directly, resulting in faster training speeds and enhanced model performance. Unlike other boosting methods, CatBoost generates symmetric trees, which reduces prediction time, improves accuracy, and mitigates overfitting through regularization. Additionally, CatBoost can handle various types of features, including numerical, categorical, and text data.

### 2.2 Stacking algorithm

Stacking is a hierarchical ensemble learning algorithm that constructs a more powerful integrated model (Meharie et al., 2022) by stacking multiple base learners with a meta-layer learner. Unlike Bagging and Boosting, Stacking focuses on optimizing the fusion of predictions from different models to achieve higher accuracy.

Instead of using a single model, we can combine the predictions from the multiple models on the basis of majority of voting for each of the output class. The voting algorithm makes more accurate final predictions by synthesizing the results of multiple independent models. Usually, the voting algorithm operates in hard voting and soft voting. As hard voting, each base model independently submits a single prediction, and the class with the highest votes is ultimately accepted as the unified conclusion. In contrast, soft voting employs a probability fusion strategy. The stacking algorithm operates as follows:

**Step1.** Divide the dataset into training set *A* and testing set *B.*
**Step2.** Perform five-fold cross-validation on the m base learners: split training set A into five mutually exclusive subsets of similar size. Sequentially select one subset as the validation set and the remaining four as the training set. Train the base learners and generate validation set predictions $A_{1}$ , $A_{2}$ , ⋯, $A_{m}$ . After fitting each base learner, generate test set predictions $B_{1}$ , $B_{2}$ , ⋯, $B_{m}$ from testing set *B.*
**Step3.** Use the validation set predictions $A_{1}$ , $A_{2}$ , ⋯, $A_{m}$ as the training set for the meta-learner. After fitting the meta-learner, feed the test set predictions $B_{1}$ , $B_{2}$ , ⋯, $B_{m}$ into the meta-learner to produce the final prediction outcome.

**FIGURE 1:**
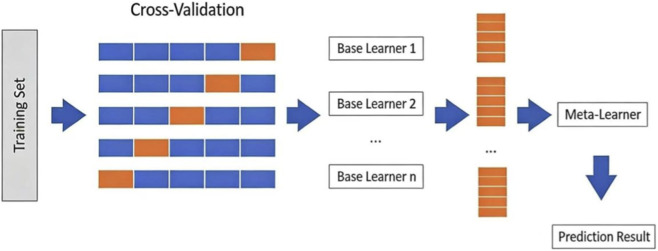
Stacking method flowchart.

The flowchart of the Stacking algorithm is shown in Figure 1.

Our proposed stacking model consists of four classifiers: DT, NB, GB and CatBoost. It is difficult to handle high dimensional data with DT and NB works well with high dimensional data due to its naive assumptions. GB has an overfitting due to target leakage problem and CatBoost can handle this problem by using several permutations of the training dataset. So, the stack of these classifiers gives more accurate results. The step of our proposed stacking model is given in Table 1.

**TABLE 1:** Stacking ensemble algorithm for heart disease prediction.

**Input: Dataset** D **, Number of folds** K
**Output: Trained stacking model** S
1 Split the dataset D into K folds
2 for each fold from 1 to K do
3 Split the dataset into training set **D** ^**K**^_**train**_ and validation set **D** ^**K**^_**val**_
4 for each base learner B_i_ in B do
5 Base model training and prediction
6 if B_i_ is Naïve Bayes then
7 Calculate prior probability for each class in **D** ^**K**^_**train**_
8 Calculate likelihood of features given in each class
9 Use Bayes theorem to calculate posterior probabilities for **D** ^**K**^_**val**_
10 Assign class labels based on probabilities to each instance in **D** ^**K**^_**val**_
11 Get prediction $P_{i}^{K}$
12 else if $B_{i}$ is Categorial boosting (CatBoost) then
13 Initialize CatBoost model with default parameters
14 Train model on **D** ^**K**^_**train**_ using boosting iteration
15 For each instance in **D** ^**K**^_**val**_, compute weighted sum of trees
16 Apply sigmoid function to get probabilities
17 Assign class labels based on probabilities
18 Get prediction $P_{i}^{k}$
19 else if $B_{i}$ is decision tree then
20 Build decision tree using training set **D** ^**K**^_**train**_ by selecting best feature
21 Prone tree to avoid overfitting
22 For each instance in **D** ^**K**^_**val**,_ traverse tree from root to leaf based on feature values
23 Assign class labels at leaf node
24 Get prediction $P_{i}^{k}$
25 end if
26 end for
27 Concatenate predictions $P^{k}$ = { $P_{1}^{k}$ , $P_{2}^{k}$ ,…, $P_{n}^{k}$ }
28 Meta model training
29 Use concatenated prediction $P^{k}$ as input features
30 Train meta learner M (gradient boosting) on $P^{k}$
31 Get meta model prediction $M^{k}$
32 end for
33 return final predictions $M^{k}$

### 2.3 SHAP

SHAP is a key methodology for interpreting machine learning model predictions. Its theoretical foundation stems from mathematician Shapleys value allocation theory in cooperative game theory, which uses Shapley values to quantify each participants marginal contribution to total payoff and determine their respective shares. In machine learning model interpretation, model predictions are treated as total payoff, with each feature acting as a participant. The Shapley value measures each features contribution to the prediction. The mathematical formula for Shapley values is provided in Equation 1.

$$\Phi_{j}\left(val\right)=\sum_{S\subseteq N\left\{j\right\}}\frac{\left|S\right|!\left(\left|N\right|‐1‐\left|S\right|\right)!}{\left|N\right|!}\left(val\left(S\cup \left\{j\right\}\right)‐val\left(s\right)\right)$$ (1)

The core principle of SHAP is to quantify each features marginal contribution to the models predictions, then compute a weighted average of these contributions to interpret the models output. The SHAP calculation process is as follows:

1. Generate all possible feature subsets by combinatorial arrangements of all features in the feature space.
2. For each feature, calculate its marginal contribution across different feature combinations, i.e., the difference in model predictions when the feature is included *versus* when it is excluded.
3. Calculate SHAP value of a feature by weighting and averaging its marginal contribution across all feature combinations.

SHAP interprets individual sample predictions to reveal why the model made specific predictions, offering detailed explanations for each sample point. By aggregating SHAP values across multiple samples, it can identify overall feature importance, delivering comprehensive model explanations. SHAP values visually demonstrate each features impact on predictions, making decision-making processes in complex black-box models like neural networks and ensemble learning more transparent and understandable, thereby enhancing model trust.

## 3 Experiment results

### 3.1 Datasets

This research utilizes the U.S. Behavioral Risk Factor Surveillance System (BRFSS) data, which systematically collected American citizens’ health information through telephone interviews in 2020. The officially preprocessed dataset of key personal indicators from the 2020 BRFSS was downloaded from Kaggle (https://www.kaggle.com/datasets). The dataset consists of 319,795 samples, 17 variables, and 1 target variable. The analysis shows that among the 17 variables, there is 1 continuous variable, 3 discrete variables, 9 binary variables, 2 multi-class nominal variables, and 2 multi-class ordinal variables. The details of the dataset variables are shown in Table 2.

**TABLE 2:** Description of variables of the dataset.

Variable name	Variable explain	Type of variable
HeartDisease	Do you have heart disease	Target variable
BMI	Body mass index	Continuous variable
Smoking	Smoking status	Binary variable
AlcoholDrinking	Drinking	Binary variable
Stroke	Stroke	Binary variable
PhysicalHealth	Number of days with physical discomfort in last 30 days	Discrete variable
MentalHealth	Number of days with psychological issues in last 30 days	Discrete variable
Sex	Sex	Binary variable
AgeCategory	Age category	Multicategorical ordered variable
Race	Race	Multicategorical nominal variable
Diabetic	Diabetes mellitus	Multicategorical nominal variable
PhysicalActivity	Have you exercised in the last 30 days	Binary variable
GenHealth	Overall health status	Multicategorical ordered variable
SleepTime	Average sleep duration	Discrete variable
Asthma	Asthma	Binary variable
KidneyDisease	KD	Binary variable
SkinCancer	Cutaneum carcinoma	Binary variable

### 3.2 Data preprocessing

#### 3.2.1 Feature encoding

The dataset comprises 17 categorical variables and 1 categorical target variable, all requiring encoding. The target variable heart disease has two categories: Yes (indicating heart disease) and No (indicating no heart disease). Categorical variables include AgeCategory, Race (American Indian/Alaskan Native, Asian, Black, Hispanic, White, Other), Diabetic (No, No (borderline diabetes),GenHealth (Excellent, Very good, Good, Fair, Poor), and Sex (Female and Male). The categorical variables Smoking, Alcohol Drinking, Physical Activity, Stroke, Skin Cancer, Asthma, Kidney Disease, and Diff Walking all have two categories: Yes and No, representing yes and no respectively.

We employ distinct encoding schemes for different variable types to preserve data structure characteristics and enhance model performance. For ordered multinomial variables, the ordinal relationships between categories significantly impact model performance and interpretability, necessitating their retention. Therefore, label encoding is applied to these variables, mapping categories to an ordered numerical scale. The GenHealth variables values (Poor, Fair, Good, Very good, Excellent) are sequentially coded as 0–4. The Age Category variables 13 age brackets are coded in ascending order as 0–12. For nominal multinomial variables without ordinal or hierarchical distinctions, label encoding is adopted to minimize additional dimensionality. This approach is particularly suitable for unordered variables where ordinal relationships have minimal impact, as distance-based machine learning models are not utilized. The Diabetic variables values (No, No (borderline diabetes), Yes, Yes (during pregnancy)) are coded as 0–4. The Race variables values (American Indian/Alaskan Native, Asian, Black, Hispanic, White, Other) are assigned numerical values from 0 to 5. For binary variables with only two possible values, this manuscript applies one-hot encoding and removes redundant feature columns. The variable Sex is coded as 1 for male and 0 for female. The categorical variables Smoking, Alcohol Drinking, Stroke, Difficulty Walking, Physical Activity, Asthma, Kidney Disease, and Skin Cancer, along with the target variable heart disease, are coded as 1 for yes and 0 for no.

#### 3.2.2 Data cleaning

The dataset used in this research contains no missing values, thus no treatment is required for them. While some features exhibit outliers, these are retained given the physiological individual variations among samples. Numerical data undergo normalization to eliminate unit and dimensionality effects.

#### 3.2.3 Data visualization analysis

**FIGURE 2:**
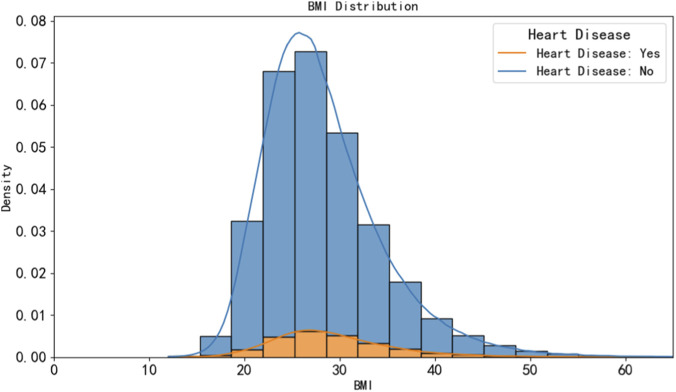
BMI stacked column chart.

The distribution of the continuous variable BMI in the dataset. As shown in Figure 2, most participants in the dataset have a body mass index (BMI) between 18 and 34. Those with lower BMI tend to have fewer heart disease cases, while the obese group with a BMI between 28 and 24 shows a higher incidence of heart disease.

The data reveals that approximately two-fifths of participants in this research are smokers, while three-fifths are non-smokers. HD patients show a higher prevalence of smoking compared to non-smokers. Regarding alcohol consumption, about one in ten individuals drink, whereas nine in ten abstain, with fewer heart disease patients consuming alcohol. Approximately one in five individuals experience walking difficulties, compared to four in five who do not. Gender-wise, the male-to-female ratio in the dataset is roughly 10:9, with slightly more male heart disease patients.

Then we employs Pearson correlation (Bonett and Wright, 2000) analysis to assess the correlation between variables, as mathematically expressed in Equation 2.

$$\rho =\frac{cov\left(X,Y\right)}{\sigma_{X}\sigma_{Y}}=\frac{E\left(\left(X-\mu_{X}\right)\left(Y-\mu_{Y}\right)\right)}{\sigma_{X}\sigma_{Y}}=\frac{E\left(XY\right)-E\left(X\right)E\left(Y\right)}{\sqrt{E\left(X^{2}\right)-E^{2}\left(X\right)}\sqrt{E\left(Y^{2}\right)-E^{2}\left(Y\right)}}$$ (2)

**FIGURE 3:**
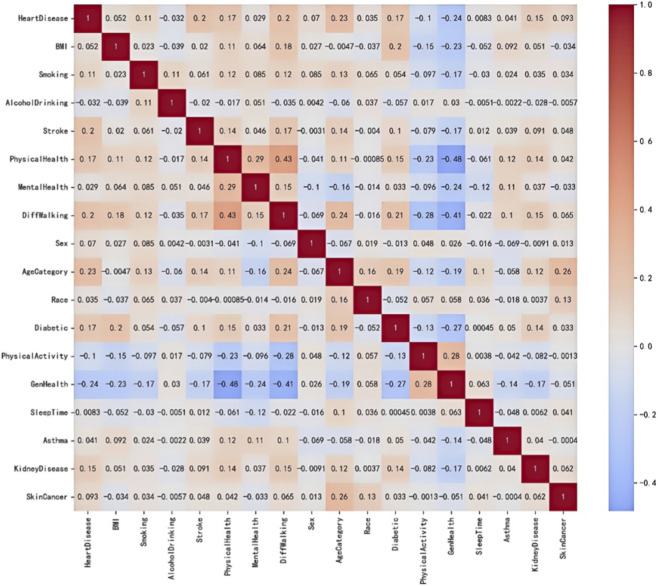
Correlation Coefficient Heat Map The heat map reveals strong positive correlations between AgeCategory, Stroke, DiffWalking, PhysicalHealth, and Diabetic, while GenHealth shows a strong negative correlation with heart disease.

The numerical characteristics of this statistic carry clear interpretive significance: positive coefficients indicate a trend of co-directional movement between variables, while negative coefficients reflect inverse correlations, with the absolute values directly proportional to the strength of the linear relationship. As shown in Figure 3, this correlation is visually demonstrated through heat maps.

#### 3.2.4 Feature selection

Proper feature selection can reduce dimensionality, improve model training efficiency, and enhance generalization capabilities. The feature selection results in this manuscript should simultaneously meet the requirements of each base learner in the Stacking integration model. Therefore, we employ three feature importance evaluation methods based on Naive Bayes, built-in feature importance in decision tree models, and built-in feature importance in CatBoost models to identify the common key features shared by these three base learners.

**FIGURE 4:**
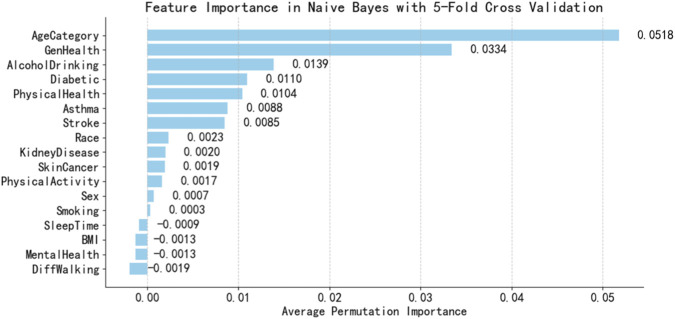
Importance ranking of permutation features based on NB.

**FIGURE 5:**
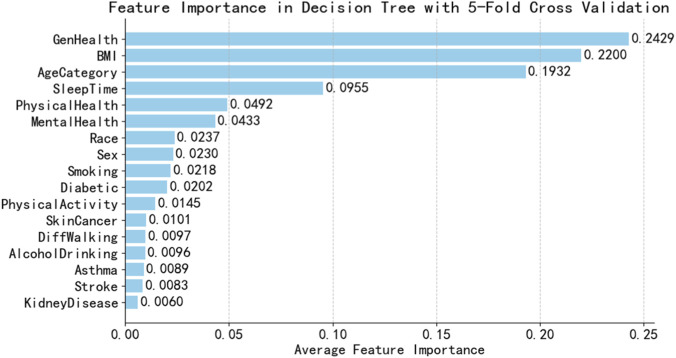
Feature importance ranking diagram based on decision tree.

This research calculated the average permutation-based feature importance of Naive Bayes model and DT model using five-fold cross-validation, with results shown in Figures 4, 5.

It is found that the feature importance ranking is different of the two models. NB is a probabilistic model, and its top feature is AgeCategory with feature importance 0.0518. While DT are tree-based models and its top feature is GenHealth with feature importance 0.2429. Uniform feature selection may remove sensitive features of some learners, thereby weakening their advantages. The decision to use the original feature space for training enables differentiated modeling through the base learner’s self-driven feature selection mechanism, thereby enhancing its diversity.

### 3.3 Evaluation indicators

A confusion matrix is a tool for evaluating the performance of classification models, presenting the comparison between predicted and actual categories in a tabular format. For binary classification tasks, the composition of a confusion matrix is shown in Table 3.

**TABLE 3:** Confusion matrix.

Actual condition	Positive prediction	Negative prediction
Actual is positive	TP	FN
Actual value is negative	FP	TN

Accuracy measures the proportion of correctly predicted samples relative to the total sample size. When positive and negative samples are nearly equal in a dataset, accuracy effectively reflects the models overall performance. However, it becomes unreliable in cases of severe class imbalance. The calculation method for accuracy is shown in Formula 3 (Zhang et al., 2023).

$$Accuracy=\frac{TP+TN}{TP+FN+FP+TN}$$ (3)

In medical diagnosis, where a single incorrect prediction can have severe consequences, high precision is particularly crucial. The precision rate is calculated as shown in Formula 4:

$$Precision=\frac{TP}{TP+FP}$$ (4)

Recall rate, also known as true rate or recall, measures the proportion of positive samples correctly predicted by the model from the actual positive class. It focuses on how many positive samples the model can retrieve. In medical diagnosis, recall rate is a crucial metric. The calculation method is shown in Formula 5:

$$Recall=\frac{TP}{TP+FN}$$ (5)

### 3.4 Parameter selection

To mitigate overfitting, this study employs 5-fold cross-validation and grid search to optimize parameters in the DT Classifier. The evaluation metrics include accuracy, precision, recall, and F1 score, with accuracy as the primary metric. The optimized parameters are presented in Table 4.

**TABLE 4:** Classifier optimal parameter of DT.

Parameter	Optimal value	Illustration
Criterion	Gini	Measure the partition quality of tree model
max_depth	1,000	Maximum depth of a tree
max_features	1,000	Number of features finding the best segmentation
min_samples_leaf	2	Minimum sample size required at leaf nodes
min_samples_split	2	Minimum sample size required for internal nodes

We employs F1 score as the objective function for Bayesian optimization (Sebastiani and Wynn, 2000), aiming to achieve an optimal balance between precision and recall. The optimal parameter values obtained by the GB model through Bayesian optimization are presented in Table 5.

The optimal parameter values obtained by the GB model through Bayesian optimization are presented in Table 6.

**TABLE 5:** Optimal parameters of CatBoost model.

Parameter	Optimal value	Illustration
Depth	8	Maximum depth of a tree
learning_rate	0.2994	Learning rate
l2_leaf_reg	4	L2 regularization coefficient
border_count	64	Number of boxes
Iterations	700	Maximum number of trees
random_strength	9.8764	Randomness of tree node splitting
colsample_bylevel	0.9188	Proportion of features in each tree
loss_function	Logloss	Loss function
random_seed	42	Random_seed

**TABLE 6:** Optimal parameters of GB.

Parameter	Optimal value	Illustration
learning_rate	0.2890	Learning rate
Loss	Exponential	Loss function
max_depth	3	Maximum depth of a single decision tree
max_features	None	Feature numbers
max_leaf_nodes	51	Maximum numbers
min_impurity_decrease	0.0989	Threshold of splitting
min_samples_leaf	2	Minimum sample size
min_samples_split	13	Minimum sample size required for a leaf node
min_weight_fraction_leaf	0.000125	Minimum weighted score
n_estimators	500	Number of trees
Subsample	0.683128	Sample proportion for fitting a single tree
validation_fraction	0.1459	Proportion of validation set partitioned in training data

### 3.5 Model comparison and analysis

We use the Borderline-SMOTE algorithm for data balancing. This algorithm considers that the classification plane is determined by the samples on the classification boundary, and thus only a few minority class samples in the boundary region are needed to generate new minority class samples. The data set is divided into training set and test set in the ratio of 8:2, and the over sampling is carried out on the training set.

**FIGURE 6:**
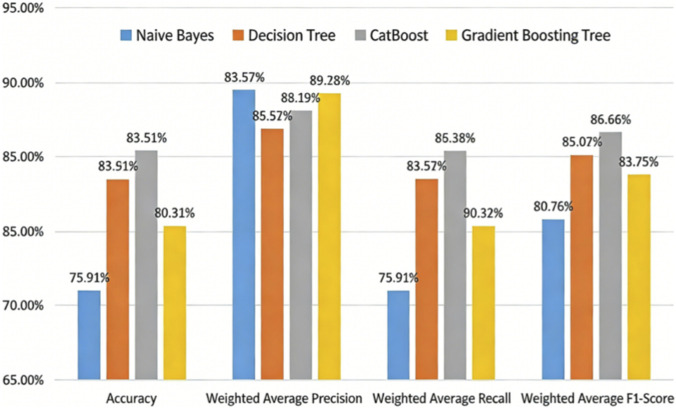
Performance of Borderline-SMOTE across different models.

From Figure 6, Borderline-SMOTE outperforms well in both Naive Bayes and decision tree models. It prevents noise amplification during sampling, thereby reducing noise-induced model bias. The Borderline-SMOTE algorithm is ultimately employed for data balancing. We selects the best combination of four models, Naive Bayes (NB), Decision Tree (DT), CatBoost and Gradient Boosting Tree (GBDT), and compares the performance of the four models on the same data set to select the best combination of Stacking integration model.

**FIGURE 7:**
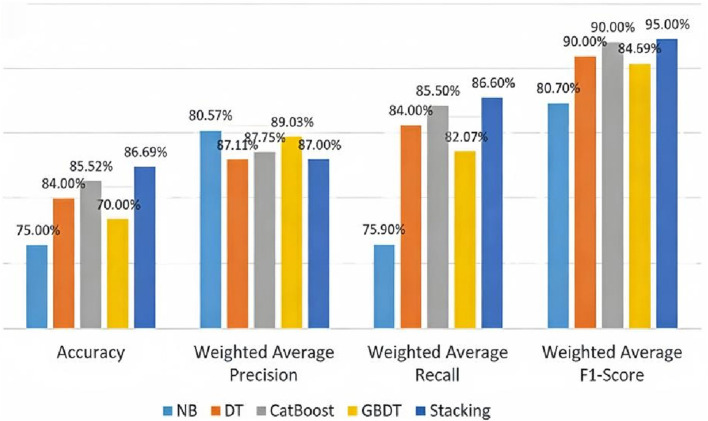
Performance comparison of stacking integrated model and single learner.

In Table 7, the base learners—probabilistic NB, rule-based DT and CatBoost, which excels at handling categorical features, not only diversify the base learners but also provide model complementarity. As a meta-learner, GB can integrate the prediction results of base learners through gradient boosting to capture complex nonlinear relationships. Further we compares the performance of a Stacking ensemble model with four single learners trained on the same dataset on the same test set, as shown in Figure 7.

**TABLE 7:** Comparison of performance of different combinations of stacking ensemble models.

Classifier	Accuracy	F1-score	Precision	Recall	Time(s)	AUC-ROC	TPR
NB	0.74	0.73	0.74	0.73	1	0.81	0.73
CatBoost	0.88	0.88	0.88	0.88	122	0.96	0.88
DT	0.88	0.88	0.87	0.89	7	0.88	0.89
GB	0.84	0.84	0.86	0.8	83	0.92	0.86
**NDCG**	0.87	0.87	0.87	0.87	455	0.97	0.89
GNCD	0.83	0.83	0.84	0.82	1,005	0.83	0.82
Hard voting	0.87	0.86	0.88	0.85	182	0.87	0.85
Soft voting	0.88	0.88	0.86	0.91	187	0.88	0.91

The performance of Stacking model in accuracy, recall and F1 score is better than that of single learner, which indicates that Stacking model can comprehensively consider the correctness of classification, the ability to identify the positive class, and the balance between accuracy and recall, and has better overall performance, which can better meet the application needs of actual medical diagnosis scenarios.

**FIGURE 8:**
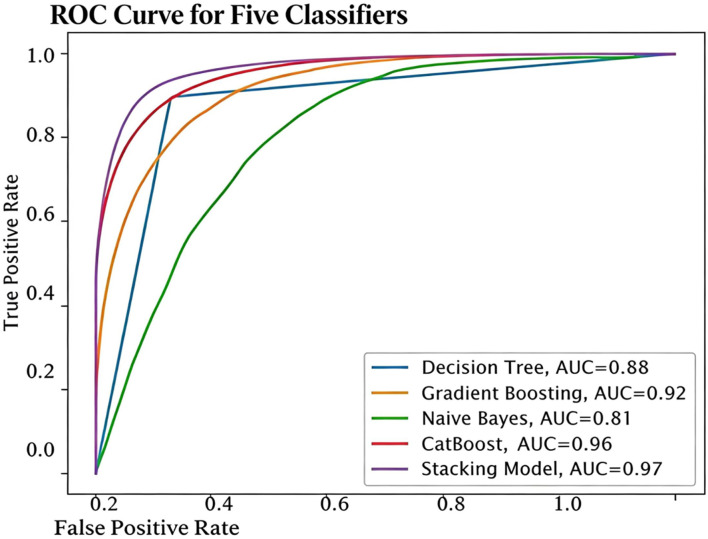
AUC-ROC of stacking model and base classifiers for heart disease prediction.

The AUC-ROC of the proposed model and base classifiers are shown in Figure 8. It clearly explains that our proposed model has the ability to better identify the TPR while minimizing the FPR.

## 4 SHAP analysis

### 4.1 Interpretability analysis

**FIGURE 9:**
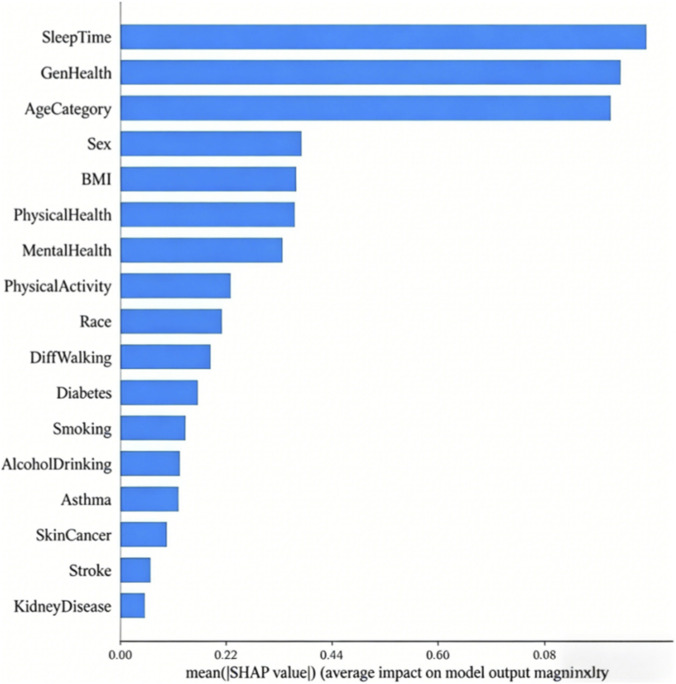
Feature importance ranking diagram based on SHAP values.

This research calculates the average SHAP values of individual features using the Stacking integration model and ranks them by numerical magnitude. Features with higher rankings demonstrate greater predictive contribution to the model. The feature ranking diagram assists physicians in identifying key factors that trigger heart disease, thereby enabling more targeted treatment strategies. As shown in Figure 9, SleepTime (average sleep duration), GenHealth (physical health status), AgeCategory (age), Sex (gender), and BMI (body mass index) rank prominently, indicating their significant reference value for heart disease diagnosis.

**FIGURE 10:**
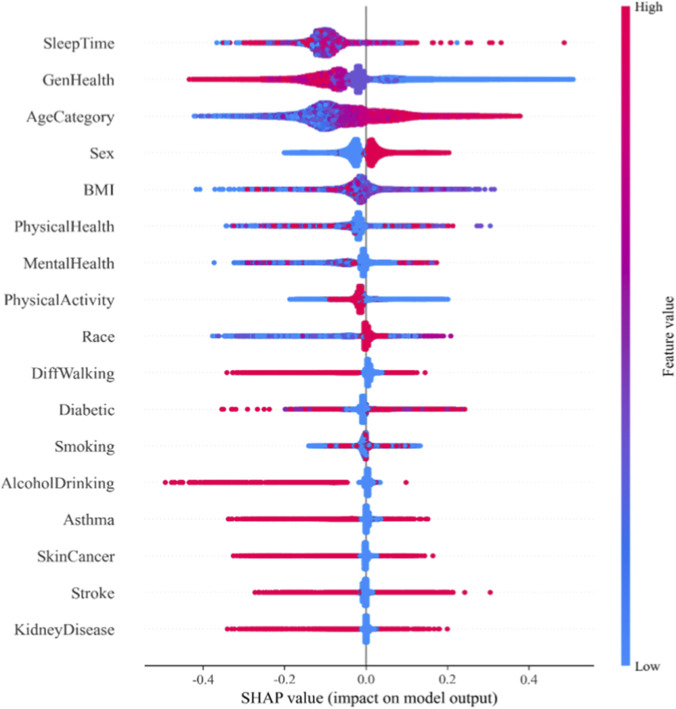
SHAP scatter plot.

While the SHAP value-based feature importance ranking diagram visually demonstrates each feature’s contribution to model predictions, it fails to capture how feature magnitude influences outcomes. This research presents a scatter plot-based summary diagram from the SHAP library to illustrate the impact of feature magnitude on test set samples’ predictions. The summary diagram is shown in Figure 10.

The vertical axis displays feature variables for heart disease prediction, sorted by average SHAP values. The horizontal axis shows SHAP values, indicating each feature’s contribution to prediction outcomes. Each point represents a sample: red indicates higher feature values, while blue indicates lower values. Points positioned further to the right demonstrate greater influence on predicting heart disease, whereas those on the left contribute more to predicting no heart disease.

Sleep Time is the most significant factor in model predictions. Moderate sleep duration (7–8 h per night) significantly reduces the risk of heart disease, whereas both excessive and insufficient sleep may increase this risk. A foreign research (Ali et al., 2020) on sleep patterns and cardiovascular disease found that chronic sleep deprivation (less than 6 h) substantially elevates heart disease risk. Sleep deprivation induces a stress response, activating the sympathetic nervous system, which accelerates heart rate, raises blood pressure, and constricts blood vessels—ultimately overloading the heart and potentially triggering or worsening heart disease.

### 4.2 Interpretability analysis of feature interactions

Many factors alone do not directly cause diseases, but when combined with multiple other factors, they may increase the risk of disease development. This research employs SHAP library’s interaction plots to investigate how the combined effects of features influence heart disease prediction outcomes. The feature interaction plot is a three-dimensional scatter diagram where the horizontal axis represents the feature value of the first feature, and the left vertical axis indicates the SHAP score of the first feature. The color of the points reflects the magnitude of the second feature’s value—bluer indicates smaller values, while redder signifies larger values.

**FIGURE 11:**
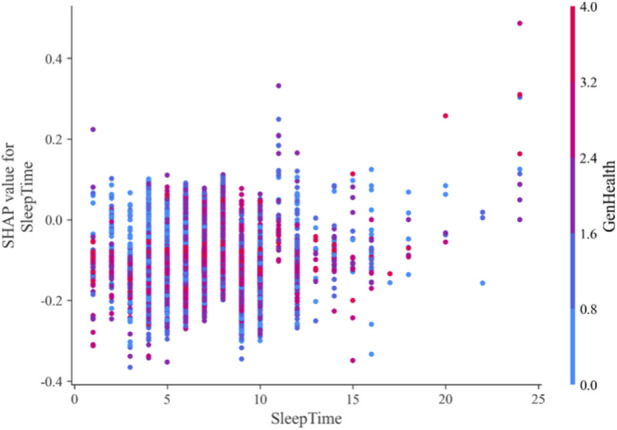
Feature interaction of SleepTime-GenHealth.

Figure 11 illustrates the interaction between average sleep duration and heart disease risk prediction. When sleep is maintained at 7–8 h, the risk of heart disease remains relatively low regardless of physical health status. Conversely, when sleep is less than 5 h, individuals with good health have a lower risk, while those with poor health experience increased risk due to sleep deprivation. This suggests that people with suboptimal health should prioritize adequate rest to reduce disease susceptibility.

**FIGURE 12:**
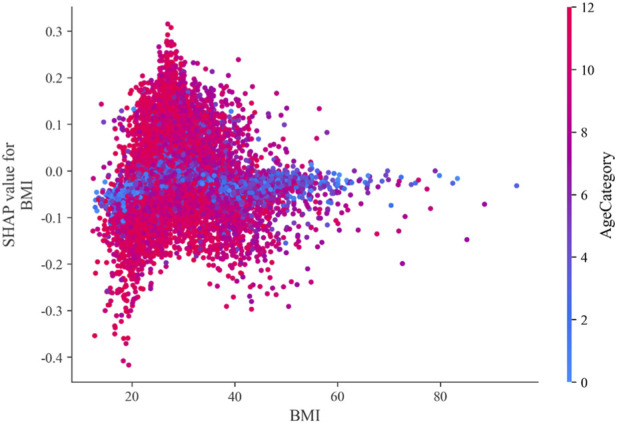
BMI-AgeCategory feature interaction.

We illustrate the interaction between body mass index (BMI) and age in predicting Heart disease and give the BMI-AgeCategory feature interaction figure in Figure 12.

**FIGURE 13:**
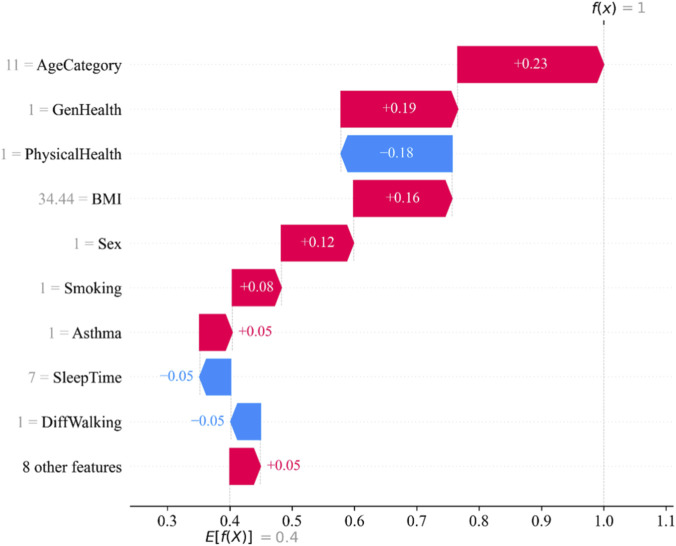
Waterfall plot of affected samples.

Further, we give the waterfall map (Figure 13) in the SHAP library visually demonstrates how a single sample’s prediction results are progressively generated by various features. By breaking down the model’s predicted values, it shows each feature’s contribution direction and magnitude to the final prediction, making the model’s decision logic clearer.

## 5 Conclusion

This manuscript presents an ensemble stacking model for heart disease prediction. Before passing data to our proposed model, we perform preprocessing by first converting string columns into integers using factorization method. Then we use Border- LineSMOTE data balancing techniques to tackle data class imbalance problem. Our proposed stacking model consists of four classifiers having three classifiers: DT, NB and CatBoost at base layer and GB at meta layer. Also we perform soft voting algorithm and compared the results with the stacking model. SHAP is used for model interpretation. Different evaluation metrices are used for validation of our proposed model. Moreover, simulations are performed and our proposed model outperforms all the base classifiers and achieved 86.69% Accuracy, 86.91% F1 Score, 87.14% Precision, 86.69% Recall, 97%, AUC-ROC, 91% TPR and 455 s execution time. In the interpretive analysis, this study employ SHAP techniques to reveal the impact of key features on prediction outcomes. Global interpretive analysis demonstrates that age, sleep duration, self-rated health status, and BMI are critical factors in assessing cardiovascular risk. Considering individual variability across samples, local interpretive analysis is conducted to evaluate the contribution of each feature to the prediction results of individual samples. In the analysis of feature interactions, the study finds that when the average sleep duration is less than five hours, individuals with better physical health have a relatively lower risk of developing heart disease, whereas those with poorer physical health exhibit a relatively higher risk.

## Data Availability

The original contributions presented in the study are included in the article/supplementary material, further inquiries can be directed to the corresponding authors.
